# Kolb, integration and the messiness of workplace learning

**DOI:** 10.1007/s40037-017-0344-2

**Published:** 2017-03-20

**Authors:** Tim J. Wilkinson

**Affiliations:** Medical Education Unit, University of Otago, Christchurch, New Zealand

‘I don’t know what’s wrong with our students. We’ve taught them all they need to know but they just can’t seem to remember any of it when they’re at work’. This paraphrase may be a familiar call of some teachers bemoaning how students have difficulty in applying their learning.

Linking classroom theory to workplace practice is the focus of a systematic review in this issue of Perspectives on Medical Education [1], but let’s start with a brief, and idiosyncratic, history of learning.

People have been learning from work and from experience since the earliest days of our evolution. Some time ago we must have decided it was a good idea to separate theory from practice so that we would teach the theory and that presumably would make the practice more robust. The dark days of the 19th century, factory style schooling attests to this philosophy: students in darkened classrooms being taught but not necessarily learning. Flexner had the best of intentions to link medical practice to theory but even here this was translated into doing the theory first and the practice later [2]. Then we must have realized that we needed to put the theory closer to practice and integrate.

Within medical education, integration became the next buzzword but there was potential for confusion. We realized that learning anatomy separately from physiology made it difficult for students to make the links between structure and function – so we invented horizontal integration where we combined previously separated disciplines in our teaching. Next came early clinical contact so that practice can be brought earlier into our curriculum – so called vertical integration. The next challenge is ‘upward’ vertical integration of theory into the later stages of our curricula so that theory becomes better linked to practice.

The irony here is we had integration of theory with practice before schools were created, and then we separated them and now we’re putting it all back together again. At least we’re trying to.

Kolb had useful views on learning from experience or linking theory to practice and his learning cycle has become part of most education courses [3]. It’s simple but also effective. In short, he describes a cycle whereby learners make links between theory and practice (or experience) in a number of ways. They can start with the theory and then apply this into practice. Or they can start with practice and reflect on how it might link to theory. Either way there is a cycle of initial theory preparation/briefing, experience, reflection/debriefing, modification of theory. With each cycle, and with ongoing experience and reflection, learners modify their views of the world. In short, they learn.

In our attempt to make learning more efficient and to provide more guidance and control, we’ve also invented learning outcomes – these are what we would like our learners to learn. They provide guidance to students and are generally seen as a very good thing.

Apprenticeship went through some phases too. Initially seen as a good thing where the protégé learns from the master, it then fell into disfavour, as it all seemed to be about practice without relevant theory. Within the health professions, working without theory is seen as a bad thing. The emergence of evidence-based medicine was one response to this. Another response was to suggest that apprenticeships are too uncontrolled.

We’ve now entered the next age of learning where we think workplace learning is good but we need to understand it better. We also need to link workplace-based learning more explicitly to theory. A laudable goal, and the focus of the systematic review in this issue [1].

There are many reasons why workplace learning is to be encouraged. We know that seeing the whole task helps a learner know where the component parts might fit [4]. We know that seeing role models and the actual doing of work helps frame learning, helps show what is relevant and helps in professional identity formation. We also know that learning in context makes it easier to apply that learning back into that context. Workplace learning is back and it’s here to stay.

However, workplace learning is also very messy. What is learnt is unpredictable and learning is not the prime activity as it takes second place to doing the work – in clinical settings, the patient is the focus not the student. The curriculum is not as well defined; it is more serendipitous. Learning outcomes are harder to control and predict. Sometimes learners do not feel welcomed in workplaces and this sense of alienation can inhibit learning.

What helps learning in workplaces and the linking of theory with practice? The first component of dealing with the messiness and unpredictability is to recognize it. Focusing on the process of learning, not just the outcomes, is an important first step [5]. To do this, we should explain the opportunities available but acknowledge that different learners will all have different experiences and take up different opportunities. We can’t control that, and shouldn’t try. We also need to acknowledge the social process of learning. The work of Lave and Wenger has been very influential here highlighting how a sense of belonging emerges from concepts of communities of practice and legitimate peripheral participation [6].

Linking theory to practice in the workplace is the focus of the systematic review in this issue [1] and it’s here where Kolb re-emerges because the components of effective activities seem to mirror his learning cycle [3]. The systematic review showed that effective interventions offered ‘just in time’ information prior to an experience or task, included effective briefing, provided well supervised and observed practice with immediate feedback, and followed it with time for reflection and good debriefing. This means the learner can consider how the experience links to existing learning and how that learning might then be modified so that the outcome is even better the next time it is put into practice. Deliberate supervised practice, with effective briefing and debriefing, seem core elements of the effective learning strategies that were identified.

We also see that people learn in workplaces despite us. The systematic review found no intervention was worse than control, and there were some where people learned just as well from the control group as the intervention.

Linking theory (or the classroom) to practice requires conscious application of a cycle of learning, while attending to the important social and professional identity components offered by workplace learning – the need for the learners to feel welcome, for them to have opportunities to observe the whole task, to observe role models and to have supervised opportunities for practice preceded by briefing and followed by debriefing. This structure does not imply formality. Such structure can be used in informal supporting ways. Workplaces do not respond well to imposed formality – we cannot easily control what people learn at work but we can help them recognize and use the learning opportunities, we can help them make sense of their experiences and most of all we can help them feel they are allowed to be there.
